# Application of the Small Punch Creep-Recovery Test (SPCRT) for the Estimation of Large-Amplitude Viscoelastic Properties of Polymers

**DOI:** 10.3390/ma16031179

**Published:** 2023-01-30

**Authors:** Jose Calaf-Chica, Pedro-Miguel Bravo-Díez, Mónica Preciado-Calzada, María-José García-Tárrago

**Affiliations:** Higher Polytechnic School (Campus Vena), University of Burgos, Avenida Cantabria s-n, 09006 Burgos, Spain

**Keywords:** SPCRT, SPT, small punch test, viscoelasticity, Maxwell-Wiechert model

## Abstract

The Small Punch Creep-Recovery Test (SPCRT) is a novel miniature test used to estimate the viscoelastic properties of polymers and biomaterials. The current investigation related to the SPCRT is limited to Finite Element Method (FEM) simulations and experimental tests on PVC. The aim of this investigation was focused on: (i) extending the experimental tests to other polymers with dissimilar viscoelastic properties; (ii) deepening the influence of non-linear viscoelastic properties in the estimation capabilities of the SPCRT; and (iii) developing a numerical methodology to estimate and take into account the viscoelastic recovery produced during the unloading step of compressive creep-recovery tests (CCRT) and SPCRTs. The experimental tests (CCRTs and SPCRTs) were done on polyethylene PE 500, polyoxymethylene POM C, nylon PA 6, and polytetrafluoroethylene (PTFE), with a range of creep loads, in the case of CCRTs, in the whole elastic regime and the surroundings of the yield strength of each material. The experimental results confirmed that the SPCRT was an accurate and reliable testing method for linear viscoelastic polymers. For a non-linear viscoelastic behavior, SPCRT estimated the viscoelastic properties obtained from CCRTs for creep loads near the yield strength of the polymer, which corresponded with large-amplitude viscoelastic properties in dynamic creep testing. In order to consider the viscoelastic recovery generated in the unloading step of CCRTs and SPCRTs, a Maxwell-Wiechert model with two branches was used, simulating the different steps of the experimental tests, and solving numerically the differential equation of the Maxwell-Wiechert model with the Runge-Kutta-Fehlberg (RKF) numerical method. The coefficients of the elements of the Maxwell-Wiechert model were estimated approaching the straining curve of the recovery step of the simulation with the same curve registered on each experimental test. Experimental CCRTs with different unloading times demonstrated that the use of this procedure derived in no influence of the unloading step time in the viscoelastic properties estimation.

## 1. Introduction

**Figure 1:**
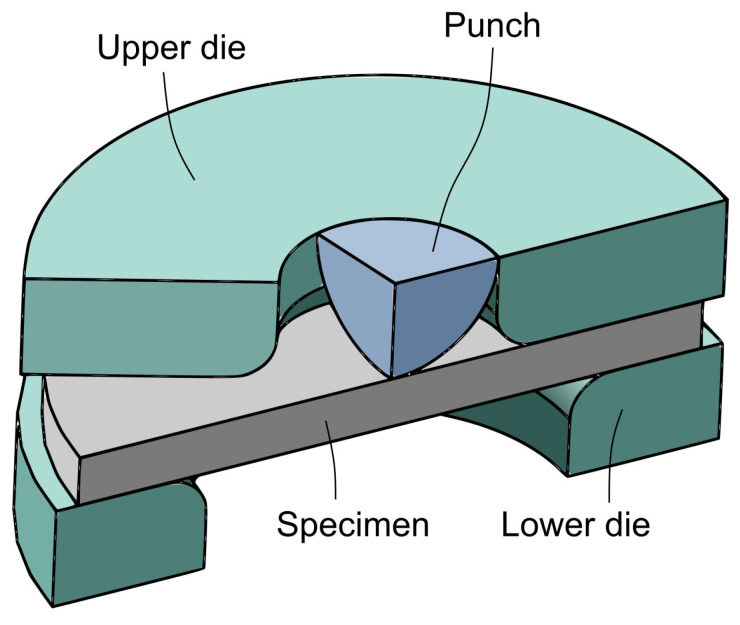
SPT set-up.

The small punch test (SPT) is a miniature testing methodology used to estimate a wide range of mechanical properties in metallic materials. From the first research developed by Manahan et al. in 1981 [1], the SPT has reached, at present, an optimum state of maturity. Standards ASTM E3205-20 [2] and BS EN 10371:2021 [3] are a clear illustration of this fact, defining and fixing the test procedure for the characterization of most common tensile properties (yield strength and ultimate tensile strength), creep and fracture toughness. Figure 1 shows the assembly of the SPT. The miniature specimen, a square of 10 × 10 × 0.5 mm or a circular plate of ≥8 mm in diameter and a thickness of 0.5 mm, is clamped between two dies and punched until failure by a sphere of 2.5 mm in diameter. The punch load versus punch displacement of this test, named as the SPT curve, is used to estimate the different mechanical properties.

The standardization of the SPT has not stopped the research effort around its applicability for: (i) the estimation of other mechanical properties, such as Young’s modulus [4], ductile-to-brittle transition temperature [5] or fatigue [6]; and (ii), the use of the SPT in non-metallic materials, such as polymers [7], biological tissues [8], and ceramics [9]. Focused on the efforts in polymers characterization, Kurtz et al. were, in 1997, the first researchers who applied the SPT in order to estimate the Young’s modulus of ultra-high-molecular-weight polyethylene (UHMWPE) [10]. Giddings et al. [11] extended this research taking the most of the miniature size of the SPT for the characterization of polymeric biomedical components in total hip replacements. The standards ASTM F2183 [12] and ASTM 2977 [13], define at present the test procedure for the estimation of mechanical properties of polymeric materials in surgical implants with the SPT. The use of the SPT for the mechanical characterization of polymeric materials still shows at this time the interest of the research community. There are illustrative examples in biomedical applications, such as testing of XL-UHMWPE polymer for use in orthopedic implants [14], estimation of mechanical properties for relevant UHMWPE formulations [15], or characterization of a PMMA-based bone cement loaded with gold nanoparticles [16]. The last years have also shown different examples of the use of the SPT in polymers without focusing on a specific industry. Koga et al. [17] evaluated with the SPT the degradation of PVC used as an electric insulation material for the electric cable. In 2021, Zhang et al. [18] obtained the correlation equations for the estimation of the yield strength of polymeric materials with the SPT. Failure behavior of polymeric membranes [19], analysis of temperature dependence of high-density polyethylene [20], or characterization of creep deformation of polymer membranes [21], are other examples of the potential of the SPT as a mechanical characterization test for polymers.

All these research lines focused on mechanical properties already investigated in the past for metallic alloys, such as elastic and plastic properties, fracture toughness, creep at high temperatures, fatigue, etc. But there was an absence of research on the applicability of the SPT for the characterization of an inherent mechanical property of polymers and biomaterials: viscoelasticity. Time-dependent viscoelastic behavior can be estimated with static (constant loading) or dynamic (cyclic loading) creep testing methodologies [22], and both of them can be correlated [23,24]. In static creep-recovery testing, a constant creep load is applied to the specimen during a prescribed time, after which loading is removed and the strain recovery is measured and registered. Depending on the testing set-up, the creep load can be in tensile, compressive, bending, or shearing modes, as can also be selected in the standard ISO 6721 [25] for the determination of dynamic mechanical properties of plastics. In 2021, Calaf-Chica et al. [26] developed the Small Punch Creep-Recovery Test (SPCRT), as a derived miniature test from the SPT, for the estimation of viscoelastic properties of polymers and biomaterials. The stress field in the SPT specimen is a complex scenario of a combination of plate bending and indentation loadings. Thus, the investigation performed by Calaf-Chica et al. was sought to demonstrate the capability of the SPT to be modified and adapted as a creep-recovery test for viscoelastic properties estimation. The miniature size of the SPT specimen provided the capability to analyze the viscoelastic behavior of polymers when there is a limited volume of available material.

**Figure 2:**
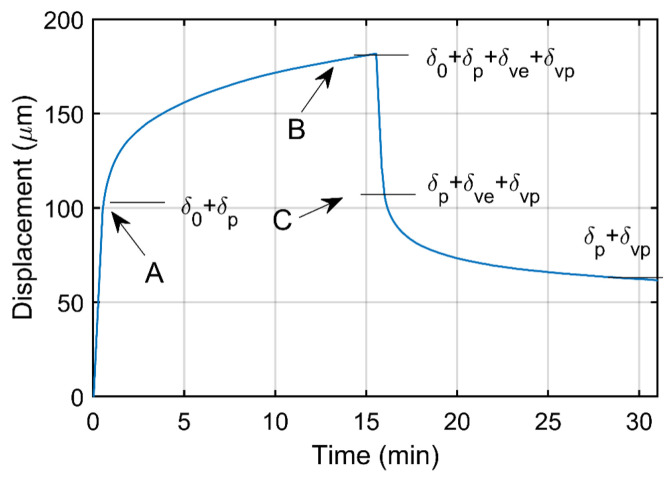
SPCRT curve: punch displacement versus time.

The set-up of SPCRT is similar to the SPT, but changing the testing steps to adapt it to a typical creep-recovery test [27]: (i) firstly, the specimen is punched with a controlled straining until reaching a punch displacement of 0.10 mm (loading step); (ii) the reached load at this displacement is fixed during a prescribed time (creep step); (iii) the specimen is unloaded with a controlled displacement (unloading step); and (iv), the time-dependent displacement recovery is registered in the absence of loading (recovery step). Figure 2 shows the punch displacement evolution along test time. The specimen is subjected at each step to different stress and strain fields: (i: loading step) non-homogeneous and time-independent elastic and plastic strainings; (ii: creep step) elastic and plastic strainings remain stable with the appearance and monotonic increasing of viscous straining, with elastic and plastic components; (iii: unloading step) the elastic deformation is recovered, and the specimen shows fields of plastic straining, and viscoelastic and viscoplastic straining; (iv: recovery step) the viscoelastic straining is recovered and, at the end of the test, the specimen only shows fields of plastic straining components (time-independent plasticity and time-dependent plasticity). The registered data for the recovery step is used to estimate the viscoelastic properties of the polymer.

The time-dependent stiffness k(t) of the SPCRT is estimated with the Equation (1), where $P_{B}$ and $\delta_{B}$ are, respectively, the punch load and the punch displacement at the beginning of the unloading step, and δ(t) is the punch displacements of the recovery step (see Figure 2).

(1) $$k\left(t\right)=\frac{P_{B}}{\delta_{B}-\delta \left(t\right)}$$

This time-dependent stiffness k(t) is dimensionless with the initial stiffness $k_{C}$ obtained from the Equation (2), $\kappa \left(t\right)=\frac{k(t)}{k_{C}}$, with κ(t) as the dimensionless time-dependent stiffness.

(2) $$k_{C}=\frac{P_{B}}{\delta_{B}-\delta_{C}}=\frac{P_{B}}{\delta_{0}}$$

Considering a viscoelastic model based on the Prony series (see Equation (3)), the Prony components, $\alpha_{i}$ (relative modulus) and $\tau_{i}$ (relaxation time), are estimated with a non-linear least squares regression, where $t_{C}$ is the initial test time of the recovery step, and *N* represents the number of Prony components.

(3) $$\kappa \left(t\right)=1-\sum_{1}^{N}\left[\alpha_{i}\left(1-e^{-\frac{t-t_{C}}{\tau_{i}}}\right)\right]$$

As previously mentioned, the SPT and the SPCRT shows a complex and non- homogeneous stress field. Thus, the SPCRT estimates a single set of Prony components for a wide range of creep stresses. This means that the SPCRT should be only applied for polymers or biomaterials with a linear viscoelastic behavior, independent of creep stress level. The experimental validation with PVC performed by Calaf-Chica et al. [26] included compressive creep-recovery tests (CCRT) with different creep loads and SPCRTs. CCRTs showed that the established creep load influenced on the estimated Prony components. Thus, PVC exhibited a non-linear viscoelastic behavior with a dependency on the creep stress level. The use of the SPCRT in this specific case estimated intermediate values of the Prony components obtained from the CCRTs. The aim of the current investigation was to extend the experimental tests to other polymers, and deepen the influence of non-linear viscoelastic behavior in the estimation capabilities of the SPCRT. In that sense, and based on the conclusions and future work derived from [26], the SPCRT could be a good testing methodology in order to estimate the viscoelastic properties derived from CCRTs with creep loads near the yield strength, and being equivalent to viscoelastic properties derived from large-amplitude dynamic creep testing. This investigation also evaluated the influence of the viscoelastic recovery produced during the unloading step of CCRTs and SPCRTs.

## 2. Materials and Methods

The sense of this investigation, as mentioned in the previous section, was to extend the experimental SPCRTs performed in previous research [26] to other polymers due to two main reasons: (i) the experimental tests are today limited to PVC, and (ii) PVC showed in that experiments a significant change in their viscoelastic properties when the CCRT was performed with a creep load near the plateau stress. This non-linear behavior affected the SPCRT estimation, obtaining higher viscoelastic properties in comparison with the CCRT estimation. Thus, this investigation selected a set of thermoplastic polymers that showed a range of viscoelastic properties in order to verify the estimating capabilities of the SPCRT. First of all, specimens based on polyoxymethylene POM C provided a significant linear viscoelastic behavior with a limited viscous component. Nylon PA 6 and polyethylene PE 500 tend to show higher levels of the viscous component with linear viscoelastic behavior. The reason for selecting these two polymers with approximately similar viscoelastic behavior was that they show too different yield and ultimate tensile strengths. Finally, polytetrafluoroethylene (PTFE) was selected as a thermoplastic with a significant non-linear viscoelastic behavior. This last material was selected in order to show if the SPCRT estimation for non-linear viscoelastic polymers provided similar results to the predicted ones for the non-linear viscoelastic behavior of PVC in the investigation performed in [26].

Compressive creep-recovery tests with different creep loads and SPCRTs were performed for each material at controlled room temperature of 21 ± 1 °C. For the CCRTs, cylindrical specimens were machined with a height of 10.0 mm and the as-built diameter. The SPCRT specimens were machined with the geometry established in the Introduction chapter. The CCRT specimens were also used to perform standard quasi-static compressive tests, in order to estimate the stress-strain curve of each material.

The CCRTs followed the next steps: (i) controlled displacement until reaching a prescribed load $P_{0}$, (ii) the load $P_{0}$ is held for 15 min, and (iii) $P_{0}$ is removed and the recovery displacement is registered during 15 min. Different prescribed loads $P_{0}$ (see Table 1) were used to evaluate the influence of the load amplitude in the viscoelastic recovery of each polymer. A range of five values for the $P_{0}$ was selected, covering the whole elastic regime that was limited by the yield strength with an offset of 0.5%. These yield strengths were estimated with quasi-static compressive tests with controlled displacement of a rate of 0.5 mm/min, and specimens with similar geometry of the CCRT specimens: 10 mm in diameter and height. For the specific case of POM C, a sixth value of $P_{0}$ over its yield strength was also analyzed in order to verify that its linear viscoelastic behavior remained linear for creep stresses over the yield strength. POM C was the polymer with the most similar viscoelastic behavior in comparison with the PVC results of [26] for the creep stresses up to the yield strength. Thus, the case with the creep load over the yield strength was used to verify if POM C also provided a sharp non-linearity in this extreme creep stress scenario.

**Figure 3:**
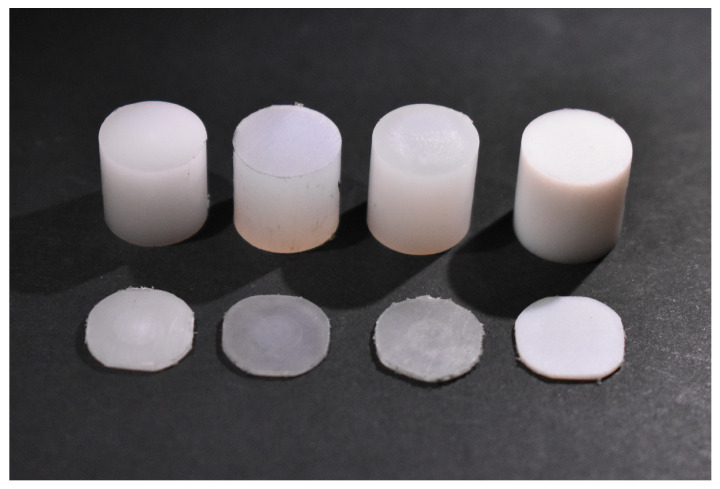
CCRT and SPCRT specimens, from left to right: POM C, PA 6, PE 500 and PTFE.

**Figure 4:**
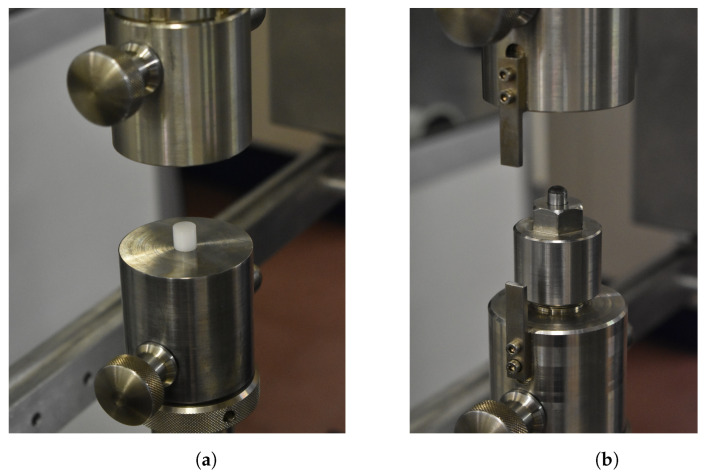
(**a**) Compressive tests and CCRTs, (**b**) SPCRTs.

Compressive tests, CCRTs and SPCRTs were performed in an electromechanical creep testing machine Zwick-Roell Kappa 050 DS. Figure 3 shows the CCRT and SPCRT specimens, and set-ups of CCRTs and SPCRTs are represented in Figure 4.

The registered displacement data from the CCRTs is dimensionless by the initial specimen height (obtaining the engineering strain ϵ(t)), and the prescribed creep loads $P_{0}$ are divided by the initial section area to obtain the engineering stress $\sigma_{0}$. Equation (4), similar to the Equation (1) for the SPCRT, estimates the time-dependent Young’s modulus. This elastic modulus is dimensionless by the initial Young’s modulus $E_{0}$ (see Equation (5)), obtaining κ(t) with Equation (6).

(4) $$E\left(t\right)=\frac{\sigma_{0}}{\epsilon_{B}-\epsilon \left(t\right)}$$

(5) $$E_{0}=\frac{\sigma_{0}}{\epsilon_{B}-\epsilon_{C}}=\frac{\sigma_{0}}{\epsilon_{0}}$$

(6) $$\kappa \left(t\right)=\frac{E(t)}{E_{0}}$$

**Figure 5:**
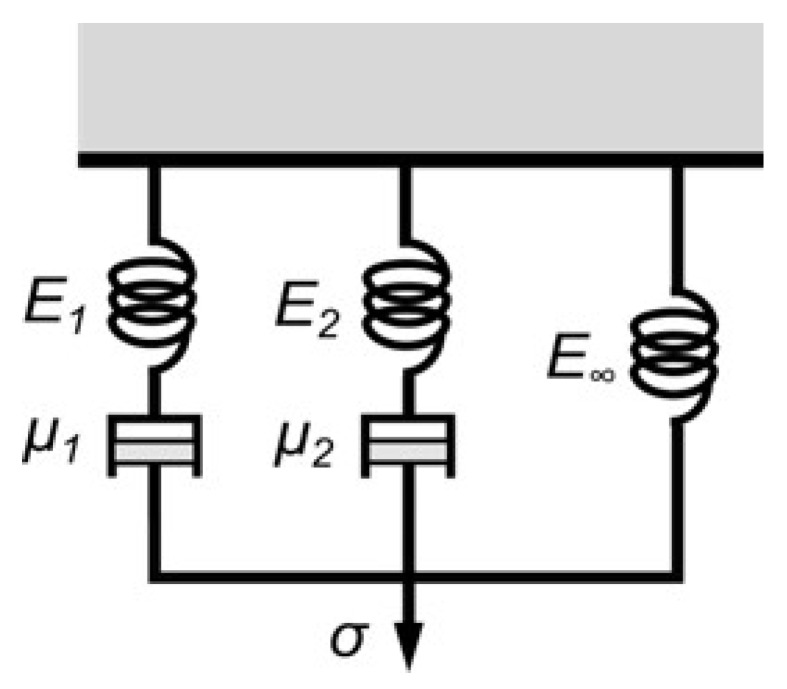
A Maxwell-Wiechert model with two branches (N=2).

The time taken in the unloading step is critical in creep-recovery tests, because the viscoelastic recovery is just initiated when the creep load begins to decline. An ideal unloading step should take non-significant time or, at least, an unloading time several orders of magnitude below the relaxation times $\tau_{i}$. In most cases, these required unloading times are unfeasible. A way to estimate the viscoelastic displacements generated during the unloading step is the analysis of a differential equation that could govern the viscoelastic behavior of the evaluated polymers. The most general model of a linear viscoelastic material is the Maxwell-Wiechert model, also known as the generalized Maxwell model. This model combines in parallel, one pure elastic spring with a set of *N* Maxwell elements (a purely elastic spring in series with a purely viscous damper). This investigation used a Maxwell-Wiechert model with N=2 (see Figure 5), because it has enough capability to follow the behavior of polymers tested in this research.

Prony series is a methodology to transform a function into a series of complex exponentials, with a real part that simulates the damping effect and an imaginary part that simulates the harmonic behavior. Thus, the Prony series could represent the solution of the differential equation of a Maxwell-Wiechert model, where each Maxwell branch would be approached by a Prony component, and the single elastic spring $E_{\infty }$, would be approached by the combination of relative moduli $\alpha_{i}$. This illustrates that a Prony series approximation could be deduced by the coefficients of a defined Maxwell-Wiechert model. As consequence, this investigation used a Maxwell-Wiechert model of two branches to approach the time-dependent dimensionless relative stiffness κ(t) of each experiment, simulating each experimental step of CCRTs and SPCRTs. This makes possible to simulate and take into account the viscoelastic recovery produced during the unloading step.

Equation (7) represents the differential equation that governs a Maxwell-Wiechert model with two branches. This analytical model was initiated after the end of the loading step. Thus, the initial conditions of each step followed: the Equation (8) for the creep step and the Equation (9) for the unloading and the recovery steps. The initial first derivative of the strain shown in the Equation (9) needs the initial strain of elastic springs of each Maxwell element ($\epsilon_{1}^{\left(E\right)}$ and $\epsilon_{2}^{\left(E\right)}$). These values are obtained from the last timing of the analysis of the previous step.

(7) $$\left(\frac{\mu_{1}\mu_{2}E}{E_{1}E_{2}}\right)\frac{d^{2}\epsilon }{dt^{2}}+\left(\mu_{1}+\mu_{2}+\frac{\mu_{1}E_{\infty }}{E_{1}}+\frac{\mu_{2}E_{\infty }}{E_{2}}\right)\frac{d\epsilon }{dt}+E_{\infty }\epsilon =\left(\frac{\mu_{1}\mu_{2}}{E_{1}E_{2}}\right)\frac{d^{2}\sigma }{dt^{2}}+\left(\frac{\mu_{1}}{E_{1}}+\frac{\mu_{2}}{E_{2}}\right)\frac{d\sigma }{dt}+\sigma$$

where $E=E_{\infty }+E_{1}+E_{2}$

(8) $$\begin{array}{cc} \; & \epsilon \left(0\right)=\frac{\sigma (0)}{E}\\ \; & {\left.\frac{d\epsilon }{dt}\right|}_{t=0}=\frac{\sigma (0)}{E^{2}}\left(\frac{E_{1}^{2}}{\mu_{1}}+\frac{E_{2}^{2}}{\mu_{2}}\right) \end{array}$$

(9) $${\left.\frac{d\epsilon }{dt}\right|}_{t=t_{0}}=\frac{1}{E}\left(\dot{\sigma }+\frac{E_{1}^{2}}{\mu_{1}}\epsilon_{1}^{\left(E\right)}+\frac{E_{2}^{2}}{\mu_{2}}\epsilon_{2}^{\left(E\right)}\right)$$

This differential equation was solved numerically with the Runge-Kutta-Fehlberg (RKF) method using an iterative procedure that changes the Maxwell-Wiechert coefficients until minimizing the mean squared error with the experimental data of the recovery step. This methodology, as previously stated, allows taking into account the viscoelastic recovery produced during the unloading step of any creep-recovery test. CCRTs for PA 6 with a creep load of 500 N was tested with two different unloading times ($t_{1}=22\;s$ and $t_{2}=1\;\min\;34\;s$) in order to show the capability of this numerical method.

## 3. Results

**Figure 6:**
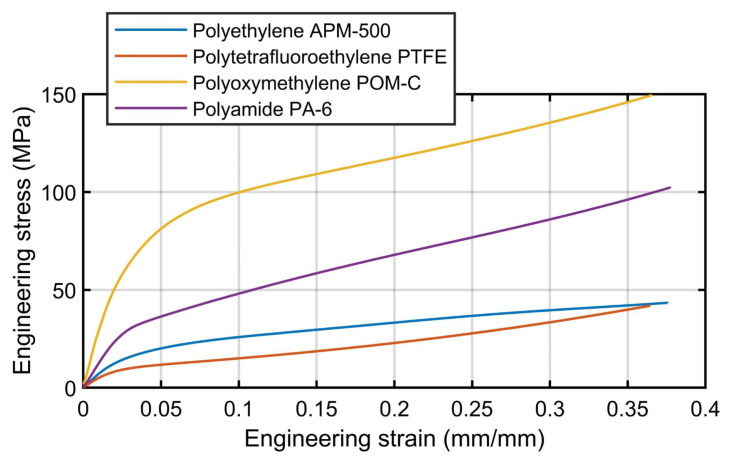
Stress vs. strain curves of the compression tests.

Figure 6 represents the engineering stress versus engineering strain curves of the compression tests, and Table 2 shows the estimated Young’s modulus and yield strength (estimated with an offset of 0.5%) of the evaluated polymers. This data was used to calculate the creep loads established in the CCRTs as previously shown in Table 1.

**Figure 7:**
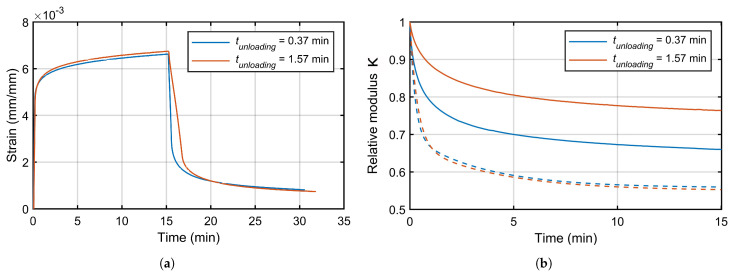
CCRTs with a creep load of 500 N (PA 6): (**a**) registered strain; (**b**) relative modulus κ(t).

Figure 7 shows the registered strain of the CCRTs of PA 6 with a creep load of 500 N and two different unloading times: $t_{1}=22\;s$ and $t_{2}=1\;\min\;34\;s$. Applying the Equations (4)–(6), the relative modulus κ(t) was estimated (see Figure 7b). The CCRT with a higher unloading time showed less viscoelastic recovery, and this is coherent with the existence of viscoelastic strain recovery during the unloading step. Both curves were used to fit numerically the coefficients of the Maxwell-Wiechert model, considering the unloading time of each test. Dashed lines of Figure 7b represent the relative modulus κ(t) showed by these Maxwell-Wiechert models applying non-significant unloading times and using the previously estimated coefficients. The similarity of both dashed curves illustrated the applicability of the Maxwell-Wiechert model in order to include the viscoelastic recovery produced during the unloading time of a creep-recovery test. These dashed lines would represent the relative modulus κ(t) estimated by an experimental creep-recovery test with an instant unloading step.

**Figure 8:**
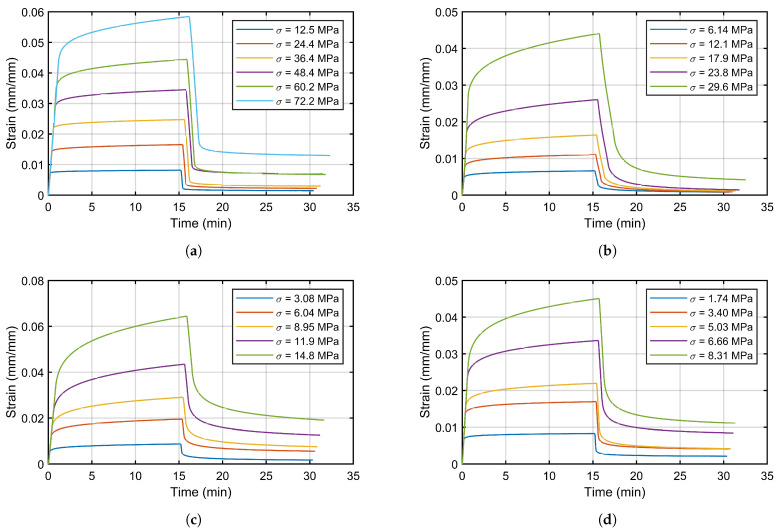
Registered strain of CCRTs: (**a**) POM C; (**b**) PA 6; (**c**) PE 500; (**d**) PTFE.

**Figure 9:**
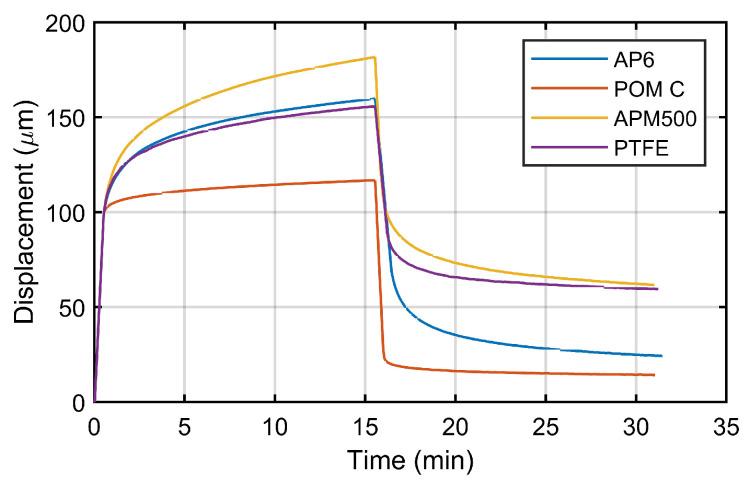
SPCRTs: registered punch displacement for the evaluated polymers.

Figure 8 represents the strain registered in CCRTs of the evaluated polymers for the creep loads $P_{0}$ established in Table 1, and Figure 9 shows the registered punch displacement of the SPCRTs for the same materials.

**Figure 10:**
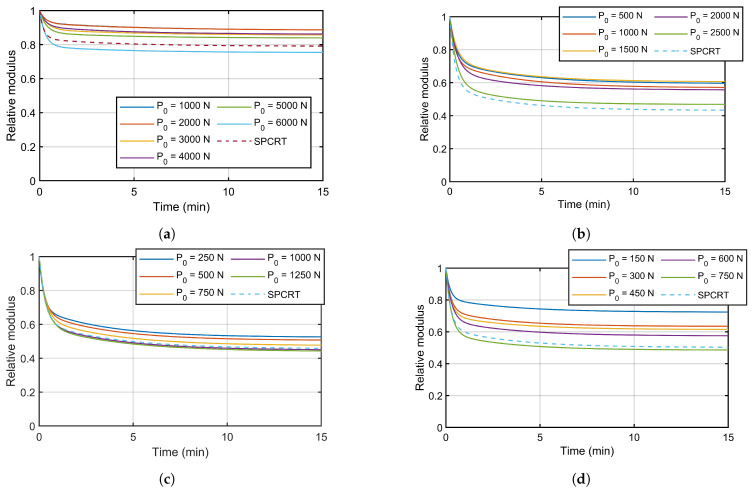
Relative modulus κ(t): (**a**) POM C, (**b**) PA 6, (**c**) PE 500, and (**d**) PTFE.

The Equations (4)–(6) were applied to the recovery step of each experimental curve, obtaining the relative modulus κ(t). A Maxwell-Wiechert model was fitted to each κ(t) experimental curve, estimating the corresponding coefficients (see Table 3). Figure 10 represents the relative modulus κ(t) for the CCRTs (smooth curves) and SPCRTs (dashed curves) generated by Maxwell-Wiechert models with non-significant unloading times and using the previously estimated coefficients. These results showed that viscoelastic properties of the evaluated materials exhibited a dependency with load $P_{0}$ established in the creep step. Thus, these polymers showed a non-linear viscoelastic behavior dependent on the stress amplitude. The SPCRTs estimated a linear viscoelastic material with a relative modulus κ(t) near the CCRTs cases with higher creep loads. Considering that these cases corresponded with creep loads near the yield strength, it means that SPCRTs estimated a linear viscoelastic model for stress amplitudes near the yield strength of the polymer.

**Figure 11:**
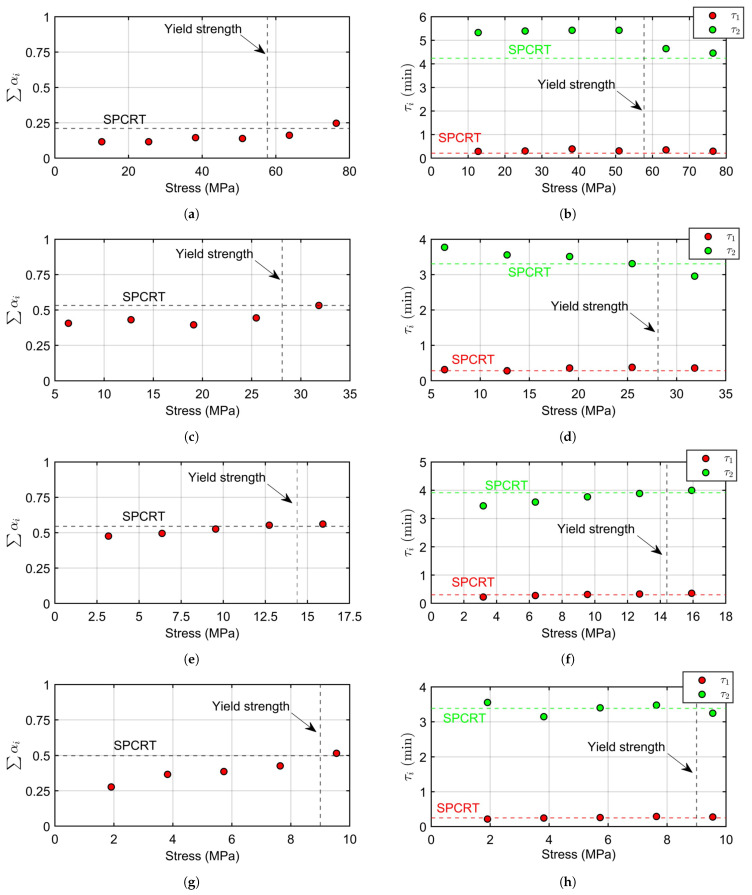
Prony coefficients for: (**a**,**b**) POM C, (**c**,**d**) PA 6, (**e**,**f**) PE 500, and (**g**,**h**) PTFE. Left graphs show the sum of relative modulus $\alpha_{i}$, and right graphs show relaxation times $\tau_{i}$.

Equation (3) and a nonlinear least squares regression were used to estimate the coefficients of a Prony series with two components (*N* = 2) that fitted the κ(t) curves of Figure 10, obtaining the results contained in Table 4. Figure 11 shows the sum of the relative moduli $\alpha_{i}$ and the relaxation times $\tau_{i}$ for each creep load of the CCRTs and each polymer. The horizontal dashed lines represent the estimated coefficients with the SPCRTs and the vertical dashed lines correspond to the yield strength of each polymer. These figures show that SPCRTs estimate the Prony components of large-amplitude elastic stresses, a fact that was also observed with the previously estimated coefficients of Maxwell-Wiechert models. The SPCRT was verified as a reliable testing procedure for the estimation of viscoelastic properties in stress amplitudes close to the yield strength of the polymer.

## 4. Discussion

The SPCRT was developed to generate an alternative testing method for estimating the viscoelastic properties of polymers when there are a limitation in the volume of the available material. The investigation performed by Calaf-Chica et al. [26] analyzed with Finite Element Method (FEM) simulations the feasibility of the estimation capability of the SPCRT. In order to support this numerical simulation, experimental tests (CCRTs and SPCRTs) were executed in a PVC polymer. In the specific case of the evaluated raw material of PVC, a low and linear viscoelastic properties were estimated with the CCRTs, except in cases with creep loads near to the plateau stress of the stress-strain curve of the polymer, where the viscous component grew dramatically. SPCRTs performed in PVC estimated viscoelastic properties with higher values of the viscous component in comparison with the estimated ones in CCRTs with creep stresses below the plateau stress. The explanation for this deviation in the estimated properties of both testing methodologies was focused on: (i) the complex and non-homogeneous stress field that shows the SPCRT specimen during the testing steps; and (ii) the sharp change in the viscoelastic properties that showed the CCRTs in PVC near the plateau stress. This assumption could not be demonstrated because CCRTs in PVC showed an unstable behavior during the creep step for creep loads over the plateau stress. In that sense, this investigation sought to extend the experimental tests performed in [26] to verify the estimating capabilities of the SPCRT.

Figure 11a,b show, for the specific case of POM C, a quasi-linear viscoelastic behavior with the lowest viscous component of the set of the evaluated polymers. CCRTs for POM C were ruled by this behavior except for the most critical creep case ($P_{0}=6000$ N) in which the viscous component grew in a 52%. The main difference between the studied case for POM C and the previously analyzed one for PVC, lies in the stable behavior of CCRTs for POM C in creep stresses over the yield strength. This allowed to estimate with the CCRTs the viscoelastic properties for a wider selection of creep stresses and the assumption performed in the conclusions of [26] was verified: Figure 11a represents as an horizontal dashed line the predicted sum of the relative moduli estimated by SPCRT for POM C, showing a value between the two estimated ones by CCRTs with the most critical creep stresses. Thus, SPCRT, which shows a non-homogeneous stress field, estimated viscoelastic properties for creep stresses close to the yield strength. Results included in Figure 11c for PA 6 and Figure 11e for PE 500, provided similar conclusions: SPCRT estimated viscoelastic properties close to the ones estimated by CCRTs with creep stresses near the yield strength of each material. These two polymers, with significantly higher viscous component, showed similar estimation capability for SPCRT in comparison with the results shown in POM C. The last material, PTFE, showed a clear non-linear viscoelastic behavior for all the creep stress levels in CCRTs, the first ones in the elastic regime and the last one just over the yield strength. This case was interesting, because the rest of the evaluated polymers showed in CCRTs an approximately linear behavior for creep stresses below the yield strength. SPCRT for PTFE estimated a sum of relative moduli similar to the ones estimated by CCRT performed with a creep stress near the yield strength. Again, the SPCRT estimated viscoelastic properties as shown for the rest of the evaluated polymers: similar to the CCRT estimation for creep stresses near the yield strength, being equivalent to the viscoelastic properties estimated from large-amplitude dynamic creep testing as reported in [23,24]. This means that SPCRT shows a high reliability and stability in their estimation capability of the viscoelastic properties of thermoplastic polymers.

As mentioned in Section 2, creep-recovery tests have a critical step, the unloading step because, at the beginning of this one, the viscoelastic recovery is initiated. This fact was demonstrated and shown in Figure 7b for CCRTs of PA 6 where depending on the time used for the unloading step, different curves of relative modulus κ(t) were estimated. Thus, the viscoelastic recovery was initiated just when the creep load began to decrease. In consequence, the use of the recovery step of the CCRT in order to estimate the viscoelastic properties would predict values less viscous than the real ones. This means that it is necessary to establish an instantaneous unloading step, in order to estimate correctly the viscoelastic properties, but this unloading step time is unfeasible. This investigation applied a numerical RKF method on the differential equation of a Maxwell-Wiechert model with two branches, in order to estimate the viscoelastic recovery generated during the unloading step of CCRTs ans SPCRTs. Figure 7b shows as dashed lines the estimated relative modulus κ(t) curves taking into account the viscoelastic straining recovery produced during the unloading step of CCRTs, using the methodology based on the Maxwell-Wiechert model. The proximity of both curves demonstrated the capability of this method in order to eliminate the requirement of “instantaneous” time for the unloading step in CCRTs and SPCRTs.

## Figures and Tables

**Table 1:** Creep loads $P_{0}$ used in the CCRTs.

Material	$P_{0}$ (N)					
POM C	1000	2000	3000	4000	5000	6000
PA 6	500	1000	1500	2000	2500	-
PE 500	250	500	750	1000	1250	-
PTFE	150	300	450	600	750	-

**Table 2:** Estimated Young’s modulus *E* and yield strength $\sigma_{y}$ from the compression tests.

Material	POM C	PA 6	PA 500	PTFE
*E* (MPa ± %)	3160 ± 4	1285 ± 2	802 ± 10	399 ± 10
$\sigma_{y}$ (MPa ± %)	57.7 ± 4.6	28.1 ± 1.7	14.4 ± 4.7	9.0 ± 4.7

**Table 3:** Coefficients of Maxwell-Wiechert model for the evaluated polymers.

Material	Test	$P_{0}$ (N)	$\sigma_{0}$ (MPa)	$E_{1}$ (MPa)	$\mu_{1}$ (MPa·s)	$E_{2}$ (MPa)	$\mu_{2}$ (MPa·s)
POM C	CCRT	1000	12.5	228	64	150	790
		2000	24.4	235	70	146	780
		3000	36.4	337	103	123	686
		4000	48.4	360	136	114	635
		5000	60.2	442	142	101	487
		6000	72.2	685	187	114	555
	SPCRT	-	-	538	105	144	615
PA 6	CCRT	500	6.14	318	95	183	645
		1000	12.1	380	97	185	613
		1500	17.9	360	120	157	530
		2000	23.8	455	160	125	435
		2500	29.6	585	192	110	385
	SPCRT	-	-	586	143	137	465
PE 500	CCRT	250	3.08	259	51	130	407
		500	6.04	280	69	125	415
		750	8.95	310	87	121	436
		1000	11.9	340	100	115	446
		1250	14.8	352	112	110	457
	SPCRT	-	-	332	89	115	445
PTFE	CCRT	150	1.74	80	16	33	114
		300	3.40	112	25	37	114
		450	5.03	124	29	33	114
		600	6.66	138	36	35	128
		750	8.31	175	42	34	124
	SPCRT	-	-	160	35	43	147

**Table 4:** Coefficients of Prony series for the evaluated polymers.

Material	Test	$P_{0}$ (N)	$\sigma_{0}$ (MPa)	$\alpha_{1}$	$\tau_{1}$ (min)	$\alpha_{2}$	$\tau_{2}$ (min)
POM C	CCRT	1000	12.5	0.071	0.288	0.045	5.328
		2000	24.4	0.073	0.306	0.043	5.393
		3000	36.4	0.111	0.391	0.034	5.424
		4000	48.4	0.093	0.309	0.046	5.424
		5000	60.2	0.130	0.351	0.032	4.646
		6000	72.2	0.210	0.295	0.037	4.457
	SPCRT	-	-	0.165	0.209	0.045	4.237
PA 6	CCRT	500	6.14	0.269	0.314	0.137	3.772
		1000	12.1	0.282	0.281	0.149	3.557
		1500	17.9	0.264	0.357	0.131	3.511
		2000	23.8	0.326	0.378	0.118	3.310
		2500	29.6	0.409	0.358	0.123	2.956
	SPCRT	-	-	0.431	0.286	0.137	3.305
PE 500	CCRT	250	3.08	0.310	0.223	0.166	3.448
		500	6.04	0.331	0.276	0.164	3.582
		750	8.95	0.362	0.314	0.164	3.766
		1000	11.9	0.393	0.330	0.160	3.886
		1250	14.8	0.404	0.356	0.157	3.996
	SPCRT	-	-	0.388	0.304	0.157	3.914
PTFE	CCRT	150	1.74	0.193	0.216	0.084	3.554
		300	3.40	0.267	0.245	0.099	3.145
		450	5.03	0.296	0.259	0.090	3.399
		600	6.66	0.326	0.290	0.100	3.477
		750	8.31	0.409	0.275	0.106	3.245
	SPCRT	-	-	0.377	0.251	0.121	3.384

## Data Availability

The data presented in this study are available on request from the corresponding authors.
